# Targeting HIF-P4H-2 in APP/PS1 Alzheimer’s mouse model improves glucose metabolism, reduces dystrophic neuritis, and maintains exploratory activity

**DOI:** 10.1016/j.jbc.2025.110432

**Published:** 2025-07-01

**Authors:** Margareta Kurkela, Lenka Dvořáková, Henna Koivisto, Maiju Uusitalo, Petri Kursula, Mikko Kettunen, Olli Gröhn, Heikki Tanila, Peppi Koivunen

**Affiliations:** 1 Biocenter Oulu, Research Unit of Extracellular Matrix and Hypoxia, Faculty of Biochemistry and Molecular Medicine, University of Oulu, Oulu, Finland; 2 A.I. Virtanen Institute for Molecular Sciences, University of Eastern Finland, Kuopio, Finland; 3 Research Unit of Protein and Structural Biology, Faculty of Biochemistry and Molecular Medicine, University of Oulu, Oulu, Finland; 4 Department of Biomedicine, University of Bergen, Bergen, Norway; 5 LINXS Institute of Advanced Neutron and X-Ray Science, Lund, Sweden

**Keywords:** Alzheimer’s disease, amyloid-beta, glucose metabolism, hypoxia, hypoxia-inducible factor

## Abstract

Alzheimer’s disease is the most common cause of dementia with limited treatment options. We asked whether activation of the hypoxia-inducible factor (HIF) pathway *via* genetic deficiency of HIF prolyl 4-hydoxylase-2 (HIF-P4H-2; also known as PHD2/EGLN1) could be an Alzheimer’s disease–modifying therapy using transgenic amyloid precursor protein (*APP*)*/*presenilin 1 (*PS1*) female mice. At 12 months of age, *APP/PS1*/*Hif-p4h-2*^gt/gt^ mice had 20% less cortical amyloid-**β** (A**β**) and less dystrophic neurites around amyloid plaques compared with *APP/PS1* mice used as controls. Compared with controls, *APP/PS1*/*Hif-p4h-2*^gt/gt^ mice were leaner, had better glucose tolerance and insulin sensitivity, and higher expression levels of an HIF target, glucose transporter 1, in the brain. These changes associated with lesser A**β** toxicity in *APP/PS1*/*Hif-p4h-2*^gt/gt^ mice linking indices of neurodegeneration with HIF-P4H-2 deficiency–mediated amelioration on brain and systemic glucose metabolism. In open field and dark–light tests, *APP/PS1*/*Hif-p4h-2*^gt/gt^ mice maintained their behavior during aging, whereas controls showed a change by 60% to 80% in exploratory activity and anxiety parameters from 6 to 12 months. Maintenance of behavior associated with cortical *Hif-p4h-2* mRNA downregulation, lesser A**β** toxicity, and lower white adipose tissue inflammation in *APP/PS1*/*Hif-p4h-2*^gt/gt^ mice. Altogether, these data connect activation of the HIF pathway *via* HIF-P4H-2 deficiency to neuroprotection in the *APP/PS1* Alzheimer’s mouse model.

Alzheimer’s disease (AD) is the most common neurodegenerative disease marked by a cognitive decline and loss of independence in daily tasks and predicted to triple worldwide by 2050 (1, 2, 3, 4). It is characterized by plaques composed of amyloid-β (Aβ) peptide, predominantly manifesting in the cerebral cortex and hippocampus, and by formation of neurofibrillary tangles (1, 2, 3, 4) of hyperphosphorylated microtubule-associated tau protein. Most AD cases appear sporadic occurring at advanced age (typically >65 years) and carrying at least one risk allele of *APOE e4*; however, now altogether, more than 40 genetic AD risk loci have been identified (3, 4). Female sex, cardiovascular and metabolic diseases, head injuries, and lifestyle and environmental factors are other risk factors for sporadic AD (4). Also, protective gene variants have been identified (3, 4). An early-onset (average ∼45 years) genetically driven dominantly inherited familial form, where the increased Aβ production is due to mutations in its precursor, amyloid precursor protein (*APP*), or APP cleaving presenilin 1 or 2 (*PSEN1*, *PSEN2*), accounts for less than 1% of the AD cases (1, 2, 3, 4). The pathophysiology of AD includes alterations in neurons, microglia, astrocytes, vessels, and glymphatic system. The accumulating Aβ and tau protein aggregates in the brain result in oxidative and inflammatory damage, which in turn leads to energy failure, synaptic dysfunction, and cell death. Despite the recent developments in the AD field, the disease represents one of the biggest unmet clinical needs (4, 5, 6).

Brain is a major consumer of oxygen and highly sensitive to alterations on O_2_ availability demonstrated by the detrimental effects of stroke. Transcriptional response of cells to hypoxia is chiefly mediated by the hypoxia-inducible factor (HIF) pathway, where three oxygen-sensing HIF prolyl 4-hydroxylases (HIF-P4Hs, also known as PHDs/EGLNs) target the HIFα subunit (HIF1α–3α) to degradation *via* von Hippel–Lindau ubiquitin ligase when O_2_ is available (7). Hypoxia inactivates HIF-P4Hs resulting in a stabilized HIFαβ dimer, which can upregulate hundreds of HIF target genes such as those regulating erythropoiesis, angiogenesis, energy metabolism, inflammatory and immunological responses, and regeneration (7). Small-molecule HIF-P4H inhibitors, which can stabilize HIF under normoxia, have recently been approved for the treatment of renal anemia (8). Preclinical data suggest that HIF-P4H inhibition could be beneficial in conditions beyond anemia, such as ischemia, metabolic syndrome, atherosclerosis, and inflammatory conditions, many of which associate with AD (9, 10).

The current literature on the HIF pathway/hypoxia in the AD pathology is limited and partly controversial. Acute and intermittent hypoxia have been associated with AD exacerbation that involves increased β-site APP–cleaving enzyme 1 (BACE1) levels, altered APP processing, induced autophagy, and increased Aβ accumulation (11, 12, 13, 14, 15, 16, 17). In contrast, hypoxic challenges have also been associated with rescued cognitive impairment, reduced Aβ load, higher brain vascularity, reduced microglial activation, and favorable APP processing (18, 19, 20, 21), whereas some studies have found no effect on APP processing (22). Overactivation of HIF1 *via* genetic means or severe hypoxia can result in dysfunction of the Aβ-associated microglia (23). On the other hand, HIF1 was associated with enhanced synaptosome and Aβ phagocytosis (24). It is conceivable that the effects of hypoxia/activation of the HIF pathway vary the current understanding associating beneficial effects to mild/moderate prolonged sustained hypoxia at any AD disease stage.

We hypothesized that genetic deficiency of the most abundant isoenzyme HIF-P4H-2 could be a novel disease-modifying therapy for AD as we have reported that *Hif-p4h-2*^gt/gt^ mice have a leaner body composition, improved glucose and lipid metabolism, less hepatic and cardiac diseases and inflammation, and larger brain capillary area, which associate with less brain protein aggregates at senescence (21, 25, 26, 27). For that, we generated a transgenic mouse line overexpressing the amyloidogenic human *APPswe* and *PSEN1dE9* variants (shortly *APP/PS1*) in the HIF-P4H-2–deficient background (*APP/PS1*/*Hif-p4h-2*^gt/gt^), whereas *APP/PS1*/*Hif-p4h-2*^wt/wt^ mice acted as controls. Female mice were followed until 12 months of age carrying out behavioral analyses from 6 months onward. Our data show that HIF-P4H-2–deficient *APP/PS1* mice expressed lower cortical Aβ load, improved glucose metabolism, which was associated with lesser Aβ neurotoxicity, and no decrease in exploratory activity compared with controls. No direct evidence of larger brain vascular area in the *APP/PS1/Hif-p4h-2*^gt/gt^ mice was detected despite larger brain glucose transporter 1 (GLUT1) area compared with controls. No difference in BACE1 levels, processing of APP, or activation of microglia was observed. Altogether, these results propose HIF-P4H-2 inhibition as a novel means to modify AD trajectory.

## Results

### HIF-P4H-2–deficient *APP/PS1* mice show normoxic activation of the HIF pathway

The *APP/PS1*/*Hif-p4h-2*^gt/gt^ mice showed similar reductions in their tissue *Hif-p4h-2* mRNA levels in comparison to *APP/PS1*/*Hif-p4h-2*^wt/wt^, which have been earlier reported for the *Hif-p4h-2*^gt/gt^ mice (21, 27, 28). The tissue with the lowest remaining levels of *Hif-p4h-2* mRNA was the heart with >90% reduction and prominent normoxic HIF1α and HIF2α stabilization, whereas the reduction in cortex was 35% and in hippocampus slightly >50% resulting in HIF2α stabilization (Fig. S1, *A* and *B*). Significantly higher mRNA levels of the HIF target mRNAs for *Glut1*, pyruvate dehydrogenase kinase 1 (*Pdk1*), and vascular endothelial growth factor a (*Vegfa*) were detected in the *APP/PS1*/*Hif-p4h-2*^gt/gt^ cortex compared with *APP/PS1*/*Hif-p4h-2*^wt/wt^ indicating activation of the HIF pathway under normoxic conditions in the former (Fig. S1*C*). Systemic activation of the HIF pathway was further supported by their 15% higher blood hemoglobin levels (Fig. S1*D*).

### HIF-P4H-2–deficient *APP/PS1* mice are protected against aging-associated obesity and tissue adiposity

**Figure 1:**
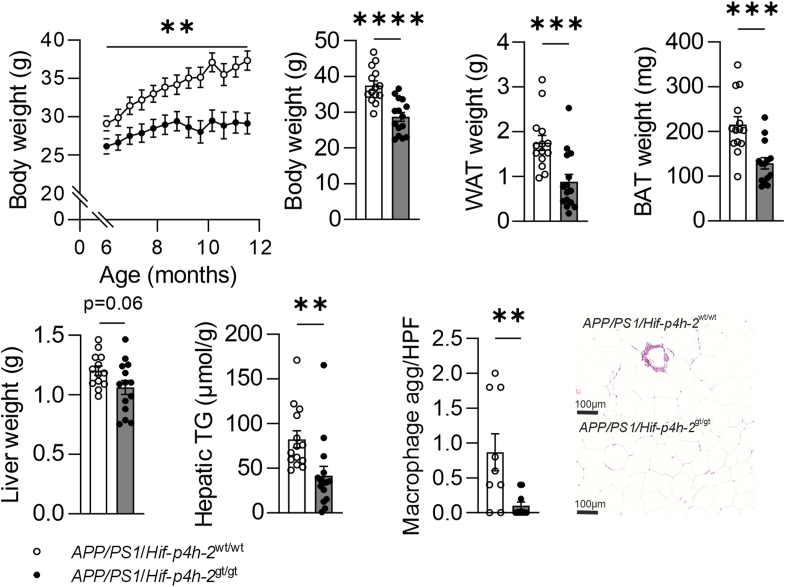
**HIF-P4H-2 deficiency reduces weight gain and adiposity in mice.** The weight gain was followed from 6 to 12 months, and the body weight, *white* adipose tissue (WAT) weight, brown adipose tissue (BAT), and liver weight in addition to the hepatic triglycerides (TGs) and the macrophage aggregates in WAT of HIF-P4H-2 wt/wt and deficient (gt/gt) *APP/PS1* mice were measured at sacrifice at 12 months of age. Data are presented as mean ± standard error of mean; ∗∗*p* < 0 0.01, ∗∗∗*p* < 0.001, and ∗∗∗∗*p* < 0.0001 in two-tailed Student’s *t* test, n = 13 to 14 wt/wt and n = 13 to 18 gt/gt. APP, amyloid precursor protein; HIF-P4H-2, hypoxia-inducible factor prolyl 4-hydoxylase-2.

The *APP/PS1*/*Hif-p4h-2*^gt/gt^ mice had a significantly lower body weight than *APP/PS1*/*Hif-p4h-2*^wt/wt^ mice during a biweekly follow-up from 6 to 12 months, the difference being ∼25% when sacrificed at 12 months (Fig. 1). The HIF-P4H-2–deficient APP/PS1 mice also had significantly less white adipose tissue (WAT), brown adipose tissue, and stored hepatic triglycerides (Fig. 1). The amount of macrophage aggregates in WAT was also significantly lower HIF-P4H-2–deficient APP/PS1 mice (Fig. 1).

### A**β** accumulation and dystrophic neurites are reduced in HIF-P4H-2–deficient *APP/PS1* brain

**Figure 2:**
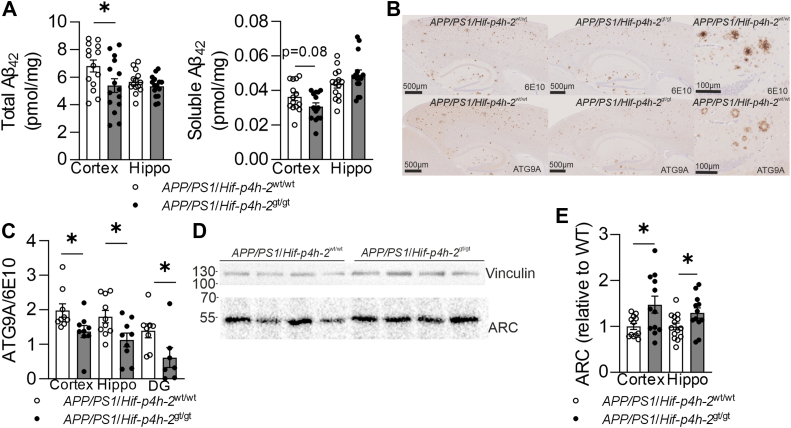
**Amyloid-β (Aβ) accumulation and neuronal toxicity are reduced by HIF-P4H-2 deficiency.***A*, total and soluble Aβ amount in the cortex of HIF-P4H-2 wt/wt and deficient (gt/gt) *APP/PS1* mice at 12 months of age. *B*, representative images of immunohistochemical staining of Aβ (6E10) and dystrophic neurites (ATG9A) to visualize dystrophic neurites and Aβ accumulation. *C*, neurotoxicity of the Aβ indicated by the ratio of ATG9A and 6E10 in cortex, entire hippocampus, and dentate gyrus (DG) of HIF-P4H-2 wt/wt and deficient (gt/gt) *APP/PS1* mice. *D*, representative Western blot of cortical ARC protein amount. *E*, ARC protein amount in cortex and hippocampus of HIF-P4H-2 wt/wt and deficient *APP/PS1* mice. Data are presented as mean ± standard error of mean; ∗*p* < 0.05 in two-tailed Student’s *t* test, n = 8 to 14 wt/wt and n = 9 to 15 gt/gt. APP, amyloid precursor protein; ARC, activity-regulated cytoskeleton–associated protein; HIF-P4H-2, hypoxia-inducible factor prolyl 4-hydoxylase-2.

When accumulation of Aβ in the 12-month-old mice was studied by ELISA, total cortical Aβ42 was reduced by 20% in the *APP/PS1*/*Hif-p4h-2*^gt/gt^ mice compared with *APP/PS1*/*Hif-p4h-2*^wt/wt^, whereas the reduction of 15% in the soluble fraction did not reach significance (Fig. 2*A*). The hippocampal Aβ levels did not differ between the genotypes (Fig. 2*A*). Despite the reduction in cortical Aβ load in the *APP/PS1*/*Hif-p4h-2*^gt/gt^ mice, there was no genotype difference in BACE1 protein or mRNA levels, processing of the APP, or activation of microglia (Figs. S2 and S3). Since the toxic effect of the Aβ, rather than the crude amyloid load, is a central phenotype of AD (29), we investigated the area of dystrophic neurites around amyloid plaques, which occur when Aβ stress impairs autophagy (30). Immunohistochemical (IHC) costainings for autophagy-related gene 9A (ATG9A) and Aβ (6E10) revealed a 30% to 70% lower ATG9A/6E10 ratio in cortex, entire hippocampus, and the dentate gyrus (DG) only in the *APP/PS1*/*Hif-p4h-2*^gt/gt^ mice compared with APP/PS1/*Hif-p4h-2*^wt/wt^ (Fig. 2, *B* and *C*). Cortical and hippocampal levels of the neural plasticity–associated activity-regulated cytoskeleton–associated protein (ARC) (31) were significantly higher in the *APP/PS1*/*Hif-p4h-2*^gt/gt^ mice than *APP/PS1*/*Hif-p4h-2*^wt/wt^ mice (Fig. 2, *D* and *E*).

### HIF-P4H-2–deficient *APP/PS1* mice have improved glucose tolerance and insulin sensitivity, which associates with neuroprotection

**Figure 3:**
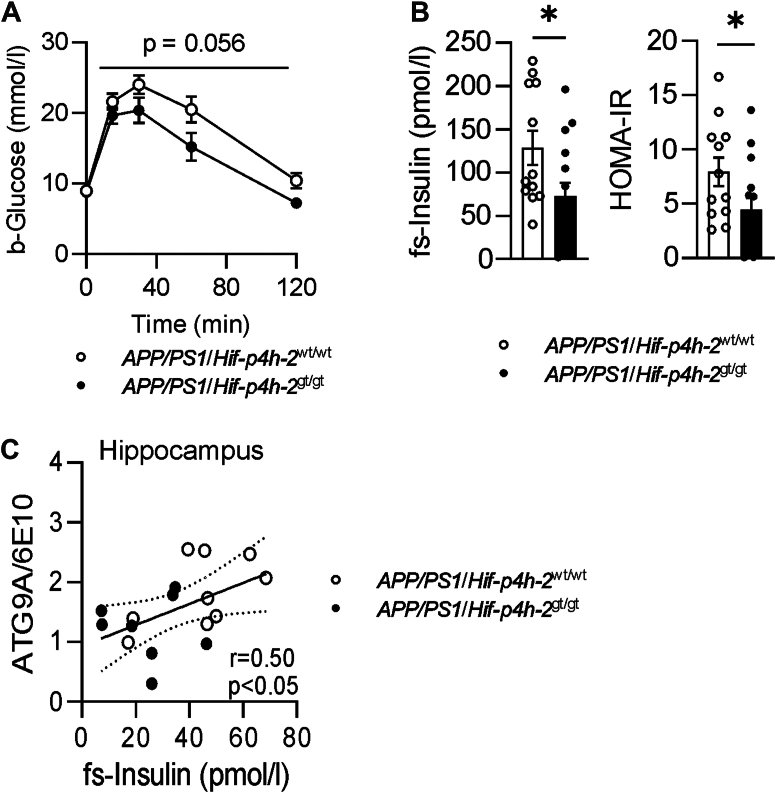
**Glucose tolerance is improved by HIF-P4H-2 deficiency in *APP/PS1* mice, and lower insulin levels associate with reduction in neurotoxicity.***A*, the glucose tolerance test of HIF-P4H-2 wt/wt and deficient (gt/gt) *APP/PS1* mice at 11 months of age. *B*, fasting serum (fs) insulin and HOMA-IR values after 3 h fasting. *C*, association of fasting insulin levels at 11 months of age with hippocampal ATG9A/6E10 ratio at 12 months of age. Data are presented as mean ± standard error of mean; ∗*p* < 0.05 in two-tailed Student’s *t* test. Pearson’s correlation was used to compare linear dependencies between two variables, n = 12 to 14 wt/wt and n = 14 to 15 gt/gt. APP, amyloid precursor protein; HIF-P4H-2, hypoxia-inducible factor prolyl 4-hydroxylase-2; HOMA-IR, homeostatic model assessment for insulin resistance.

In a glucose tolerance test performed at 11 months of age, *APP/PS1*/*Hif-p4h-2*^gt/gt^ mice showed improved glucose clearance from blood compared with *APP/PS1*/*Hif-p4h-2*^wt/wt^ (Fig. 3*A*), and they had lower fasting insulin levels and insulin resistance scores (Fig. 3*B*). Fasting insulin levels associated positively with hippocampal ATG9A/6E10 ratio (Fig. 3*C*), suggesting that the healthier glucose metabolism contributed to neuroprotection in the *APP/PS1*/*Hif-p4h-2*^gt/gt^ mice.

### GLUT1-positive area but not vascular area in the brain is increased in HIF-P4H-2–deficient *APP/PS1* mice

**Figure 4:**
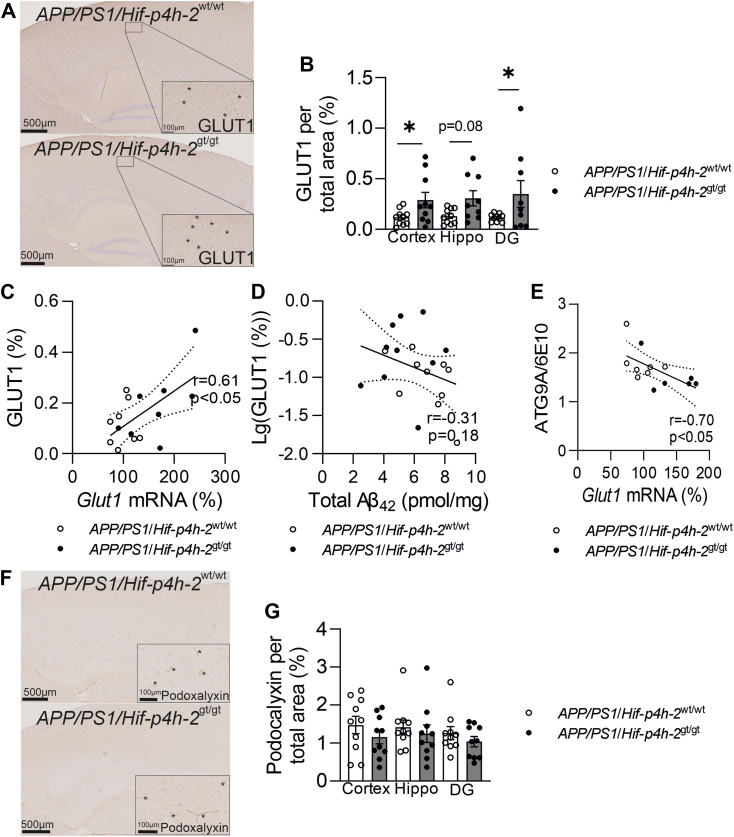
**Glucose transporter 1 (GLUT1)–positive area is increased in HIF-P4H-2–deficient *APP/PS1* mice.***A*, representative images of GLUT1 immunohistochemical staining, (*B*) GLUT1 area in cortex, entire hippocampus, and dentate gyrus (DG) of HIF-P4H-2 wt/wt and deficient (gt/gt) *APP/PS1* mice at 12 months of age. Association between (*C*) cortical GLUT1 and *Glut1* mRNA, (*D*) cortical GLUT1 and Aβ amount, and (*E*) cortical neurotoxicity and *Glut1* mRNA. *F*, representative images of podocalyxin immunohistochemical staining. *G*, podocalyxin area in cortex, entire hippocampus, and DG of HIF-P4H-2 wt/wt and gt/gt *APP/PS1* mice. Data are presented as mean ± standard error of mean; ∗*p* < 0.05 in two-tailed Student’s *t* test. Pearson’s correlation was used to compare linear dependencies between two variables, n = 8 to 14 wt/wt and n = 8 to 15 gt/gt. Aβ, amyloid-β; APP, amyloid precursor protein; HIF-P4H-2, hypoxia-inducible factor prolyl 4-hydoxylase-2.

GLUT1 is expressed at high levels selectively by brain capillary endothelium, and it has therefore been used as a marker for brain vascularity. We showed by IHC staining that the relative GLUT1-positive area was significantly larger in cortex and DG of the *APP/PS1*/*Hif-p4h-2*^gt/gt^ than *APP/PS1*/*Hif-p4h-2*^wt/wt^ mice (Fig. 4*A*). There was a strong positive correlation between the cortical protein and mRNA expression level of GLUT1 in both genotypes (Fig. 4*B*). Although no significant correlation between total Aβ42 level and GLUT1 area was detected (Fig. 4*D*), there was a strong negative correlation between cortical *Glut1* mRNA level and the ATG9A/6E10 ratio (Fig. 4*E*), suggesting that higher GLUT1 expression contributed to neuroprotection in the *APP/PS1*/*Hif-p4h-2*^gt/gt^ mice. Since *Glut1* is an HIF target gene, the detected increase in GLUT1 area may not directly indicate a larger capillary area but rather its higher expression level in a given endothelial cell, reflecting increased glucose intake. To control for that, we studied another commonly used brain vascular IHC marker, podocalyxin, expressed on the luminal membrane of brain microvascular endothelial cells. The relative podocalyxin-positive area was larger than the GLUT1-positive area in both genotypes (Fig. 4, *B* and *F*), but no difference in its area between the genotypes was detected in cortex, entire hippocampus, or DG (Fig. 4*F*).

To further assess the effect of HIF-P4H-2 deficiency on brain *in vivo*, we performed volumetric MRI as well as T_2_∗ relaxation time and perfusion (arterial spin labeling) mapping in isoflurane-sedated 2-month-old *Hif-p4h-2*^gt/gt^ and *Hif-p4h-2*^wt/wt^ mice. The imaging showed a ∼10% reduced brain size in the *Hif-p4h-2*^gt/gt^ mice compared with *Hif-p4h-2*^wt/wt^ mice (Fig. S4*A*) but no significant difference in T_2_∗ relaxation time or cerebral blood flow (CBF) in cortex or hippocampus between the unchallenged genotypes (Fig. S4, *B* and *C*).

### HIF-P4H-2–deficient *APP/PS1* mice do not show aging-associated changes in exploratory activity

**Figure 5:**
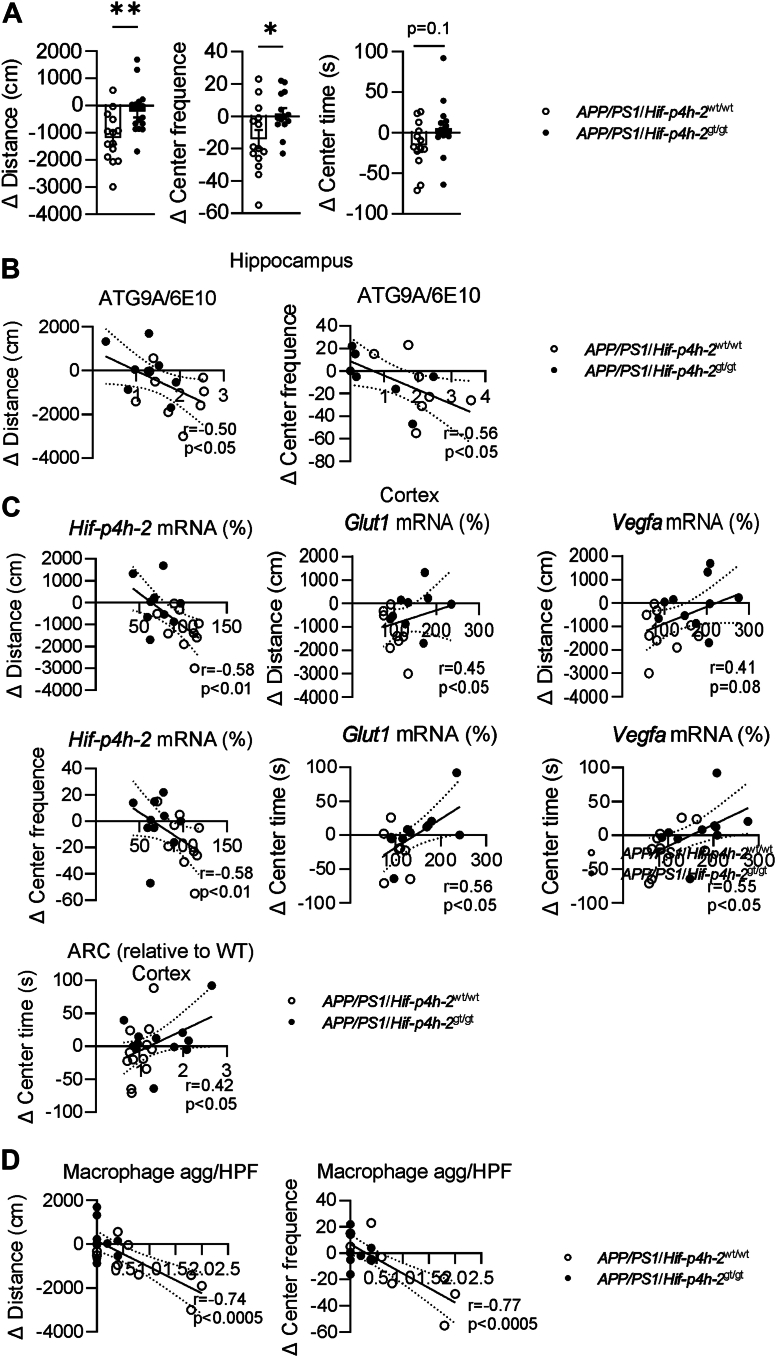
**Age-related changes in open field parameters have significant correlations with both neurodegeneration and HIF pathway activation.***A*, difference in open field activity parameters between 6 and 12 months in HIF-P4H-2 wt/wt and deficient (gt/gt) *APP/PS1* mice. *B*, association between hippocampal dystrophic neurites and difference in open field activity between the age of 6 and 12 months. *C*, association between cortical mRNA expression levels or ARC protein amount and difference in open field activity between the age of 6 and 12 months. *D*, association between WAT macrophage aggregates and difference in open field activity between the age of 6 and 12 months. Data are presented as mean ± standard error of mean; ∗*p* < 0.05, ∗∗*p* < 0.01 in two-tailed Student’s *t* test. Pearson’s correlation was used to compare linear dependencies between two variables, n = 8 to 14 wt/wt and n = 9 to 12 gt/gt. APP, amyloid precursor protein; ARC, activity-regulated cytoskeleton–associated protein; HIF-P4H-2, hypoxia-inducible factor prolyl 4-hydoxylase-2; WAT, white adipose tissue.

*APP/PS1* mice are known to have a hyperactive phenotype (32, 33). We studied spontaneous exploratory activity and fear/anxiety using open field and dark–light tests. At the age of 6 months, *APP/PS1*/*Hif-p4h-2*^wt/wt^ females had 35% higher locomotor activity than *APP/PS1*/*Hif-p4h-2*^gt/gt^ females, whereas no genotype difference was detected at 9 and 12 months of age (Fig. S5). At 6 months, *APP/PS1*/*Hif-p4h-2*^wt/wt^ mice made 45% more visits to the open field center; however, at 9 months, there was no difference, and by 12 months, they made 150% fewer visits to the open field center than *APP/PS1*/*Hif-p4h-2*^gt/gt^ mice (Fig. S5). The time spent in the open field center showed an even clearer opposite trend with age between the genotypes, such that by 12 months, the *APP/PS1*/*Hif-p4h-2*^gt/gt^ mice spent over 400% more time in the center than *APP/PS1*/*Hif-p4h-2*^wt/wt^ mice (Fig. S5). A two-way ANOVA revealed a significant genotype × age interaction for distance, center frequency, and center time (*p* < 0.0001, <0.05, and <0.05, respectively) (Fig. S5). The reduced time in the open field center in *APP/PS1*/*Hif-p4h-2*^wt/wt^ mice by age may result from a reduced exploratory drive upon repeated exposure to the same environment or increased level of anxiety for an open place with age. Correspondingly, the unaltered locomotor activity with a moderate increase in time spent in the open field center points to a maintained exploratory drive or reduced level of anxiety with age in *APP/PS1*/*Hif-p4h-2*^gt/gt^ mice. Altogether, the difference in locomotor activity and dwell time in the open field center between 6 and 12 months showed a reduction by 60% to ∼80% in the *APP/PS1*/*Hif-p4h-2*^wt/wt^ mice, whereas no significant change was detected in *APP/PS1*/*Hif-p4h-2*^gt/gt^ mice (Fig. 5*A*). There were negative correlations between the aging-associated difference in behavioral parameters and ATG9A/6E10 ratio in hippocampus (Fig. 5*B*) and in the cortical expression of *Hif-p4h-2* mRNA (Fig. 5*C*), and positive correlations to the expression of cortical *Glut1* and *Vegfa* mRNA and ARC protein levels (Fig. 5*C*), suggesting a causality between the cortical HIF-P4H-2 deficiency–activated HIF pathway, healthier neurites, and maintained behavior. Interestingly, there was also a negative correlation between aging-associated differences in behavior and WAT macrophage aggregates (Fig. 5*D*), suggesting that the lesser systemic inflammation in the *APP/PS1*/*Hif-p4h-2*^gt/gt^ mice (Fig. 1) associated with preserved exploratory activity.

**Figure 6:**
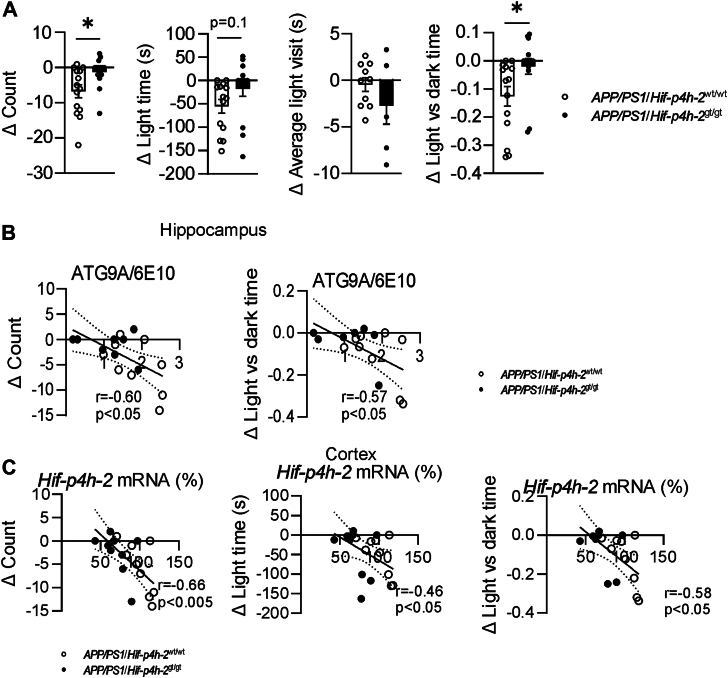
**Age-related changes in dark–light parameters have significant correlations with both neurodegeneration and *Hif-p4h-2* downregulation.***A*, difference in dark–light activity parameters between 6 and 12 months in HIF-P4H-2 wt/wt and deficient (gt/gt) *APP/PS1* mice. *B*, association between hippocampal dystrophic neurites and difference in dark–light activity between the age of 6 and 12 months. *C*, association between cortical *Hif-p4h-2* mRNA levels and difference in dark–light activity between 6 and 12 months of age. Data are presented as mean ± standard error of mean; ∗*p* < 0.05 in two-tailed Student’s *t* test. Pearson’s correlation was used to compare linear dependencies between two variables, n = 8 to 13 wt/wt and n = 8 to 13 gt/gt. APP, amyloid precursor protein; HIF-P4H-2, hypoxia-inducible factor prolyl 4-hydoxylase-2.

A similar pattern of behavioral changes between the genotypes was observed in the dark–light test performed at 6, 9, and 12 months; the *APP/PS1*/*Hif-p4h-2*^wt/wt^ mice displaying ∼70% age-dependent reduction in number of visits to the light area, total time spent in the light and the light versus dark time (Fig. S6). Interestingly, the *APP/PS1*/*Hif-p4h-2*^gt/gt^ mice showed a significant increase in their average light visit duration upon aging, whereas no such trend was seen in *APP/PS1*/*Hif-p4h-2*^wt/wt^ mice (Fig. S6). When analyzed with a two-way ANOVA, effect of genotype × age was significant for number of visit and light time (*p* < 0.01 and <0.05, respectively) (Fig. S6). This finding corroborates the finding of increased dwell time upon aging in the open field center by *APP/PS1*/*Hif-p4h-2*^gt/gt^ mice. Both observations point to a maintained exploratory drive or decreased anxiety by age. Altogether, the difference in the number of visits to light area and the ratio between the time spent in the light versus dark area between 6 and 12 months showed a reduction by >80% in *APP/PS1*/*Hif-p4h-2*^wt/wt^ mice compared with *APP/PS1*/*Hif-p4h-2*^gt/gt^ mice (Fig. 6*A*). These aging-associated behavioral differences associated negatively with hippocampal ATG9A/6E10 (Fig. 6*B*) and cortical *Hif-p4h-2* mRNA levels (Fig. 6*C*) supporting the data from the open field test.

## Discussion

We show that systemic HIF-P4H-2 deficiency, which activates the HIF pathway in normoxia, results in healthier metabolism and lesser amyloid load and toxicity in *APP/PS1* mice. Our results also suggest a causal relationship between brain HIF-P4H-2 deficiency and maintained behavioral phenotype upon aging in *APP/PS1* mice.

The detected larger GLUT1-positive brain area, which associated with higher cortical expression of *Glut1* mRNA, is likely indicative of higher glucose intake capacity of the *APP/PS1*/*Hif-p4h-2*^gt/gt^ endothelial cells compared with controls. GLUT1 is often used as a marker for brain capillary area. However, this interpretation is confounded by the fact that GLUT1 is an HIF target gene. We did not find a similar increase in the podocalyxin-positive brain area, suggesting that there was no difference in the capillary area between the *APP/PS1*/*Hif-p4h-2*^gt/gt^ mice and controls despite higher expression of cortical *Vegfa* mRNA in the former and association of its higher expression levels with stable behavior upon aging. This interpretation was further supported by MRI finding that 2-month-old *Hif-p4h-2*^gt/gt^ mice did not differ from their wildtype controls in CBF. This study did not have a separate *APP/PS1* wildtype littermate group to address whether *APP/PS1* overexpression leads to reduced glucose intake. However, an earlier study found reduced GLUT1 amount in the hippocampus but no differences in capillary density in 18-month-old *APP*/*PS1* mice (34). Thus, we can conclude that normoxic HIF activation corrected this deficit. Notably, a reduced ^18^fluorodeoxyglucose uptake in several brain regions in a PET scan is an established early sign of AD (35). If normoxic HIF activation can ameliorate this deficit, it has an obvious therapeutic potential.

Being the primary energy source of neurons, increased brain glucose intake is considered neuroprotective (36). Here, we found reduced cortical Aβ levels and a smaller area of dystrophic neurites around amyloid plaques in relation to amyloid plaque area in *APP/PS1*/*Hif-p4h-2*^gt/gt^ mice. The reduction in cortical Aβ levels was somewhat unexpected, since HIF1α upregulates both BACE1 and γ-secretase activity for Aβ production in brain acute hypoxia *in vivo* or in primary neuronal culture (37). However, *Hif-p4h-2* downregulation did not affect either APP or BACE1 protein or mRNA levels, suggesting that either its effects on the HIFα subunits or its moderate congenital downregulation induces opposite effects to acute HIF1α stabilization. Furthermore, we found no difference in the area of microglia around amyloid plaques or in plaque-free brain sections between the *APP/PS1*/*Hif-p4h-2* genotypes. These findings indicate that at least altered levels of the Aβ precursor protein or its main cleaving enzyme BACE1 cannot account for the reduced cortical amyloid load, neither increased microglial activation. More importantly, *Hif-p4h-2* downregulation proved to be neuroprotective in terms of reduced dystrophic neurites around amyloid plaques and increased levels of synaptic plasticity–associated protein ARC. The area of dystrophic neurites correlated positively with fasting serum insulin levels and negatively with hippocampal *Glut1* mRNA levels, again linking the indices of neurodegeneration with capacity for improved brain glucose uptake. Recently, the importance of HIF1α in regulating astrocytic glycolysis was highlighted in connection to AD pathology because of activation of indoleamine-2,3-dioxygenase 1 and increased kynurenine levels where indoleamine-2,3-dioxygenase 1 inhibition restored HIF-mediated glycolysis and homeostasis (38).

The indices of improved systemic and brain glucose metabolism with *Hif-p4h-2* downregulation in *APP/PS1* mice also translated into behavioral changes. Mice that were wildtype for *Hif-p4h-2* showed reduced exploratory activity with aging and repeated exposure to the test environment, especially to open and brightly lit sections. In contrast, *APP/PS1*/*Hif-p4h-2*^*gt/gt*^ mice maintained their exploratory activity across the studied ages. Interestingly, across both genotypes, the aging-related reduction in exploratory activity correlated with a larger area of dystrophic neurites in the hippocampus. This correlation is difficult to ascribe to the synaptic damage, since neurotoxic lesions of the hippocampus in rats or mice usually lead to hyperexploration, not reduced exploration in the open field (39, 40). On the other hand, both maintained exploratory activity and dwell time in the open field center across ages correlated positively with cortical *Glut1* mRNA levels. In turn, cortical *Glut1* mRNA levels correlated inversely with the area of dystrophic neurites. Collectively, these findings suggest that improved glucose uptake in the brain contributes to both reduced neuropathology and maintained exploratory behavior in *APP/PS1*/*Hif-p4h-2*^*gt/gt*^ mice.

Most prior studies on activation of the HIF pathway on AD have predominately been carried out in the *APP/PS1* mice by environmental hypoxia, which has often been very severe (down to 9% O_2_), having cycles of hypoxia and normoxia when a reperfusion injury is likely to occur, and the length of the intervention varying from 14 to 60 days (11, 12, 13, 14, 15, 16, 17). The setting here is thus very different when the HIF pathway was activated congenitally by genetic inactivation of *Hif-p4h-2*. When comparing the outcome of the *APP/PS1*/*Hif-p4h-2*^gt/gt^ mice to *APP/PS1* kept in sustained 15% O_2_ for 6 weeks from 10 months onward, no difference in the open field test was observed when compared with normoxia (21% O_2_) kept mice, even though the Aβ load in cortex and hippocampus was 20% reduced and as was their ATG9A/6E10, whereas no difference in insulin resistance was observed (21). When the *APP/PS1* were subjected to 15% O_2_ for 6 weeks at an earlier time point, 4 months of age, reduced activation of microglia, lower levels of BACE1, and higher physiological processing of APP were observed, which were not seen in the *APP/PS1*/*Hif-p4h-2*^gt/gt^ mice at 12 months here (21). As we did not analyze an earlier time point, it remains to be studied whether this is a difference because of means of activation of the HIF pathway or the disease trajectory.

Higher altitude of residence has been associated with lower dementia mortality rate, suggesting that oxygen levels might have direct long-term effects on brain physiology (41). Several small-molecule HIF-P4H inhibitors, which unselectively inhibit all three isoenzymes, have been accepted for treatment of renal anemia (8). Whether these could be used to modify AD likely depends on their ability to cross the blood–brain barrier, which appears limited (42). Considering the 35% to 50% downregulation of *Hif-p4h-2* mRNA in the *APP/PS1*/*Hif-p4h-2*^gt/gt^ brain may suggest that beneficial effects may be conveyed even with a mild brain delivery. Whether activation of the HIF pathway by HIF-P4H inhibitors in peripheral tissues, such as the key regulators of glucose metabolism and insulin signaling; liver, WAT, and skeletal muscle could contribute to AD protection remains to be studied. Roxadustat was shown recently to increase glucose intake, glycolysis, and insulin-stimulated glycogen synthesis in human skeletal muscle biopsy–originated myotubes (43). Insulin resistance and type 2 diabetes have close connections and increase the risk of sporadic AD (44). Our data here suggest that the large-scale genetic HIF-P4H-2 deficiency can prevent insulin resistance in the familial early-onset AD and the lower serum insulin levels contribute to lesser Aβ toxicity. The latter is in line with insulin resistance increasing Aβ accumulation and tau hyperphosphorylation (45, 46, 47). Moreover, the lower systemic inflammation, associated with better insulin sensitivity—characterized by a smaller number of WAT macrophage aggregates in the *APP/PS1*/*Hif-p4h-2*^gt/gt^ mice—associated with maintained exploratory behavior, suggesting a role for metabolic factors to AD phenotype even in the genetic APP/PS1 model.

The limitations of the study include tissue-level analyses being performed only at one time point (12 months) and lack of a memory test. However, data from several analyses coincide; simultaneous presence of lower Aβ_42_ load, lesser area of dystrophic neurites, higher levels of the synaptic plasticity maintaining ARC, and preserved exploratory activity across ages in the *APP/PS1/Hif-p4h-2*^gt/gt^ mice compared with controls. Another limitation is the lack of information on the knockdown level of HIF-P4H-2 in individual cell types in the brain. Therefore, we cannot conclude which cell types should (or should not) be targeted by HIF-P4H-2 inhibition for neuroprotection. However, cell-specific therapeutic delivery is still very challenging, and likely, any arising treatment could not be targeted.

In conclusion, our data provide basis for inhibiting HIF-P4H-2 in AD as a multieffect target, which decreases Aβ load and toxicity, insulin resistance, and brain hypometabolism and most importantly retains behavior.

## Experimental procedures

### Generation of mouse lines

All animal experiments were performed according to the protocols approved by the Animal Experiment Board of Finland, license numbers ESAVI-8179-04.10.07-2017 or ESAVI/35415/2021 30/2021. *Hif-p4h-2*^gt/gt^ mice had been generated using a GeneTrap targeting vector introduced into intron 1 of the *Hif-p4h-2* gene as previously described (28). *APP*swe/*PS1*dE9 (*APP/PS1*) mice were acquired from a colony at the University of Eastern Finland, Kuopio, and crossbred with *Hif-p4h-2*^wt/gt^ mice to generate *APP/PS1*/*Hif-p4h-2*^gt/gt^ female mice from heterozygous matings. The weight of the mice was followed biweekly starting from 6 months old and continued until sacrifice at the age of 12 months. Results from our previous study (21) were used for power calculations. To reach statistical significance with probability of 80%, 14 mice were needed for each group.

### Behavioral studies

The behavioral assessment of the mice was carried out at 6, 9, and 12 months of age using open field and dark–light tests (48, 49). For the open field test, mice were placed in the corner of a 30 × 40 cm open field enclosure with light transmitted through the bottom. The mice were allowed to move freely in the field for 10 min, while all movement was recorded with a Logitech Webcam C930e camera, and the recordings were analyzed using Ethnovision 16.0 software using sample rate 9.13 fps, open field area 30 × 40 cm, and inner zone 10 × 20 cm.

For the dark–light test, the 30 × 40 cm enclosure was divided into dark–light area, both with the area of 20 × 30 cm. The mouse was placed into the dark area for an adjustment period of 3 min, before a passageway with a diameter of 4 cm was opened allowing the mouse to move freely between the dark and light area. All movements in the lit area were recorded for 10 min to calculate the number and duration of visits to the lit area.

### MRI measurements

The MRI measurements were performed with the 7-T/16-cm horizontal Bruker Pharmascan system with a standard Bruker quadrature resonator volume coil and a mouse brain quadrature surface coil in 2-month-old *Hif-p4h-2*^wt/wt^ and *Hif-p4h-2*^gt/gt^ littermate female mice. The animals were anesthetized with isoflurane (in N_2_/O_2_ 70%/30%) adjusted between 1.5% and 2% to keep respiration at 60 breaths per min. The temperature of the animals was maintained at 37.5 °C with heated water circulation in the animal bed. For volumetric MRI and T_2_∗ quantification, a three-dimensional multigradient echo sequence was used with the following parameters: TR = 68 ms, TE = 2.73 ms, echo spacing 2.9 ms, echoes 13, flip angle 16°, and matrix size 125 μm^3^. For the arterial spin labeling CBF measurements, we used Flow-Sensitive Alternating Inversion Recovery EPI, TI: 35, 100, 200, 300, 400, 500, 600, 700, 800, 900, 1000, 1100, 1200, 1300, 1400, 1600, 1700, 2500, 5000, 8000 ms, matrix 96 × 64, field of view 1.92 × 1.28 cm, slice thickness 1 mm, and coronal slice at level 78 (atlas.brain-map.org). CBF measurements of 30 s were repeated four times within 30 min. Total duration of isoflurane anesthesia was 60 to 75 min. All MRI data were processed and analyzed with in-house created scripts, Snakemake (https://snakemake.github.io/) (50), Python (version 3.10, https://www.python.org/downloads/), and advanced normalization tools (http://stnava.github.io/ANTs/) (51). The individual multi-gradient echo images were registered to the anatomical template using affine and nonlinear SyN registration. The CBF maps were calculated from the change in apparent T_1_ relaxation times between global and local inversion experiments (blood T_1_ was fixed to 2.4 s) (52). The T_2_∗ maps were estimated as monoexponential decays. Regions of interest (cortex, hippocampus) were drawn on target brain and projected on individual maps using the translation matrices from the coregistration, and the mean values were estimated. To evaluate the brain size, a brain mask was drawn on the target brain image and transferred to the individual multi-gradient echo brain images. The number of voxels and the volumes in mm^3^ were computed in each mask for all individuals.

### Glucose tolerance test

The glucose tolerance of mice was assessed at 11 months. The mice were fasted for 3 h and sedated with 4 μl/g fentanyl and 1 μl/g midazolam cocktail. A blood sample from *vena saphena magna* was taken for serum analysis, and the blood glucose (Contour XT), lactate (Lactate Scout+), and hemoglobin (Triolab HemoCue Hb 201+) levels were measured. Glucose (1 mg/kg) was administered intraperitoneally to the mice, and peripheral glucose levels were measured after 15, 30, 60, and 120 min of injection.

### Sacrifice and sample collection

The mice were sacrificed at 12 months. The mice were fasted 3 h, and anesthetic cocktail (12 μl/g fentanyl and 3 μl/g midazolam, 2.25 ml/g domitor) was administered subcutaneously. Blood sample for serum analysis was collected from *vena cava*, and blood glucose, lactate, and hemoglobin levels were measured. The mouse was perfused with 0.9% ice-cold NaCl through the left ventricle of the heart using peristaltic pump for 3 min, and the organs were collected.

### Histological analyses

The formalin-fixed paraffin-embedded brain tissues were sectioned into 5 μm sagittal sections that were stained with Dako REAL EnVision Detection System (K5007; Aglinet) IHC staining kit. Aβ-positive accumulation was quantified with a human-specific anti-Aβ antibody (6E10; BioLegend). Autophagotic cells were quantified with an anti-ATG9A antibody (ab108338; abcam). Activated glial cells were quantified with an anti-IBA1 antibody (019-19741; Wako). Capillaries were quantified from 10 μm paraffin-embedded sections with an anti-GLUT1 antibody (catalog no.: 07-140; Merck) or antipodocalyxin (MAB1556; R&D Systems). The number of macrophage aggregates in the WAT was quantified from sections stained with hematoxylin–eosin. The sections were scanned with Hamamatsu NanoZoomer S60 slide scanner. The analysis of the histological samples was carried out using the Visiopharm VIS software.

### A**β** ELISA

Flash-frozen tissue pieces were homogenized in 10 μl PBS per mg tissue, and homogenate was aliquoted for analyses by ELISA, quantitative real-time PCR (qPCR), and Western blot. Protease phosphatase inhibition cocktail (04693132001; Roche) was added to the homogenized cerebral cortex and hippocampus. Total Aβ_42_ was determined by diluting tissue homogenate 1:6 in 6 M guanidine to solubilize all Aβ. The soluble fraction of Aβ_42_ was determined directly from homogenate. The concentration of both soluble and total Aβ_42_ was determined using Human/Rat Beta Amyloid (42) ELISA Kit Wako, High-Sensitive (292-64501) kit according to the manufacturer’s instructions. The final dilution factor was 1:2000 (soluble fraction) and 1:60,000 (total Aβ_42_).

### qPCR analyses

Total RNA was isolated from the sample homogenate using E.Z.N.A. Total RNA Kit (I for heart, liver, and muscle; II for cerebral cortex, hippocampus, and WAT) (Omega Bio-Tek) and reverse transcribed with an iScript cDNA synthesis Kit (Bio-Rad). qPCR was performed with iTaqSYBR Green Supermix with ROX (Bio-Rad) with the primers shown in Table S1.

### Western blot analyses

Protease phosphatase inhibitor cocktail (catalog no.: 04693132001; Roche) was added to the sample homogenate aliquot. The protein (50 μg) from cerebral cortex or hippocampus was resolved by SDS-PAGE gel, blotted, and probed by anti-BACE1 (1:500 dilution, catalog no.: PA1-757; Invitrogen), anti-β-actin (1:5000 dilution, catalog no.: NB600-501; Novus Biologicals), or ALFA-tagged anti-ARC nanobody (C11, 1 μg/ml) (53, 54, 55, 56). The protein (20 μg) was resolved by SDS-PAGE gel, blotted, and probed by anti-C-terminal APP (1:4000 dilution, catalog no.: A8717; Sigma). Nuclear extracts from protein lysates were extracted using NER-PER kit (Pierce). Nuclear extracts (20 μg) were resolved by SDS-PAGE, blotted, and probed by anti-HIF1α (1:500 dilution, catalog no.: NB100-479; Novus Biologicals) or anti-HIF2α (1:500 dilution, NB100-122; Novus Biologicals) and a horseradish peroxidase–conjugated secondary anti-rabbit or anti-mouse antibody (1:5000 dilution; DAKO) or anti-ALFA-tag (1:2000 dilution; Nano-Tag) for anti-ARC nanobody was used to detect the primary antibody. The Pierce ECL system (ThermoScientific) or Clarity Max (Bio-Rad) was used for detection of secondary antibody. Results were semiquantified using Fiji (ImageJ) software.

### Blood and serum analyses

Blood samples were collected at glucose tolerance test and sacrificed, and serum was separated by centrifugation (2000*g* at 20 min + 4 °C). Serum insulin levels were determined using kit (catalog no.: 90082; CrystalChem) according to the manufacturer’s instructions. The homeostatic model assessment for insulin resistance value was determined by calculating; (*blood glucose* [*mmol/l*] *∗ serum insulin* [*pmol/l*])*/156.65*. Serum cholesterol, triglyceride, and high-density lipoprotein levels were determined using respective reagents (catalog nos.: 04718917, 07528604, and 46219201; Roche) according to the manufacturer’s instructions. The absorbances were measured using the Tecan Spark plate reader.

### Determination of triglycerides in liver

Liver tissues were incubated in EtOH–KOH solution at 55 °C overnight and centrifuged 10,000*g* for 5 min. The triglycerides were measured from the supernatant (Roche), and absorbances were measured using the Tecan Spark plate reader.

### Statistical analyses

Student’s two-tailed *t* test or two-way ANOVA was used for analysis of statistical differences between groups. Pearson’s correlation coefficient was used to compare linear dependencies between two variables. All data are presented as mean ± SEM, and *p* < 0.05 was considered statistically significant: ∗*p* < 0.05, ∗∗*p* < 0.01, ∗∗∗*p* < 0.001, and ∗∗∗∗*p* < 0.0001.

## Data availability

All data are available in the main text or the supporting information.

## Supporting information

This article contains supporting information.

## Conflict of interest

The authors declare that they have no conflicts of interest with the contents of this article.
