# Supplementary material for: Targeting HIF-P4H-2 in APP/PS1 Alzheimer’s mouse model improves glucose metabolism, reduces dystrophic neuritis, and maintains exploratory activity
Source: J Biol Chem. 2025 Jul 1;301(8):110432. doi: 10.1016/j.jbc.2025.110432 (PMC12314380; doi:10.1016/j.jbc.2025.110432)
Supplement: Supporting information [file mmc1.pdf]

Supporting Information

Targeting HIF-P4H-2 in APP/PS1 Alzheimer's mouse model improves glucose metabolism, reduces dystrophic neurites and maintains exploratory activity

Margareta Kurkela[1], Lenka Dvořáková[2], Henna Koivisto[2], Maiju Uusitalo[3], Petri Kursula[3,4,5], Mikko Kettunen[2], Olli Gröhn[2], Heikki Tanila[2], and Peppi Koivunen[1*]

[1]Biocenter Oulu, Research Unit of extracellular matrix and hypoxia, Faculty of Biochemistry and Molecular Medicine, University of Oulu, Oulu, Finland. [2]A.I. Virtanen Institute for Molecular Sciences, University of Eastern Finland, Kuopio, Finland. [3]Research Unit of protein and structural biology, Faculty of Biochemistry and Molecular Medicine, University of Oulu, Oulu, Finland. [4]Department of Biomedicine, University of Bergen, Bergen, Norway. [5]LINXS Institute of Advanced Neutron and X-Ray Science, Lund, Sweden.

*Correspondence to: Peppi Koivunen, MD, PhD, Faculty of Biochemistry and Molecular Medicine, University of Oulu, Aapistie 7C, P.O. Box 5400, FIN-90014, Finland, Email peppi.koivunen@oulu.fi,Tel. +358-8-5375822

This PDF file includes:

Table S1

Figs. S1 to S6

Supporting Table 1. Primers used in qPCR analyses.

Gene	Forward primer (5’ → 3’)	Reverse primer (5’ → 3’)
B-2-microglobulin	GGTCTTTCTGGTGCTTGTCTCA	GTTCGGCTTCCCATTCTCC
Bace1	CAGTGGGACCACCAACCTTC	GCTGCCTTGATGGACTTGAC
B-actin	CGATGCCCTGAGGCTCTTTTC	TCTTTACGGATGTCAACGTCACACT
Glut1	TCAAACATGGAACCACCGCTA	AAGAGGCCGACAGAGAAGGAA
Hif-p4h-2	GCGTCCCAGTCTTTATTTAGATA	CTGGGCAACTACAGGATAAAC
Pdk1	AGGATCAGAAACCGGCACAAT	GTGCTGGTTGAGTAGCATTCTAA
Vegf-a	GCACTGGACCCTGGCTTTAC	AACTTGATCACTTCATGGGACTTCT

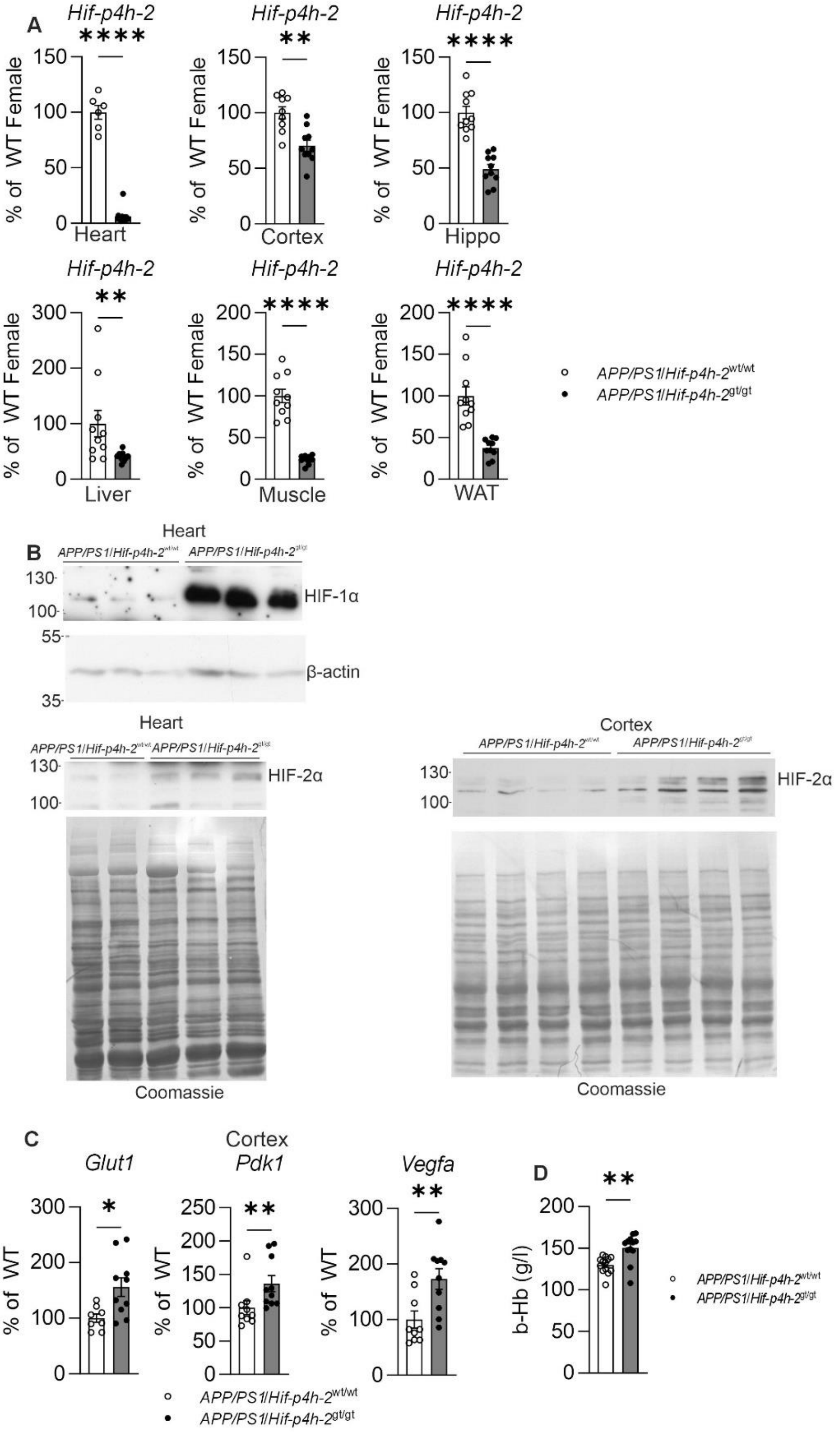

Supporting Figure 1. HIF response is activated in the HIF-P4H-2-deficient *APP/PS1* mice. (**A**) *Hif-p4h-2* mRNA expression in various tissues of 12-month-old HIF-P4H-2 wild-type (wt/wt) and deficient (gt/gt) *APP/PS1* mice. (**B**) Representative Western blots indicating normoxic stabilization of HIF1α and HIF2α in the heart and HIF2α in the cortex of HIF-P4H-2-deficient *APP/PS1* mice. (**C**) Cortical mRNA levels of a selection of HIF target genes. (**D**) Blood hemoglobin (Hb) levels in HIF-P4H-2 wild-type and deficient *APP/PS1* mice. Data are presented as mean ± standard error of mean. WT, wild type. $^*P < 0.05$, $^{**}P < 0.01$, $^{****}P < 0.0001$ in two-tailed Student's t-test.

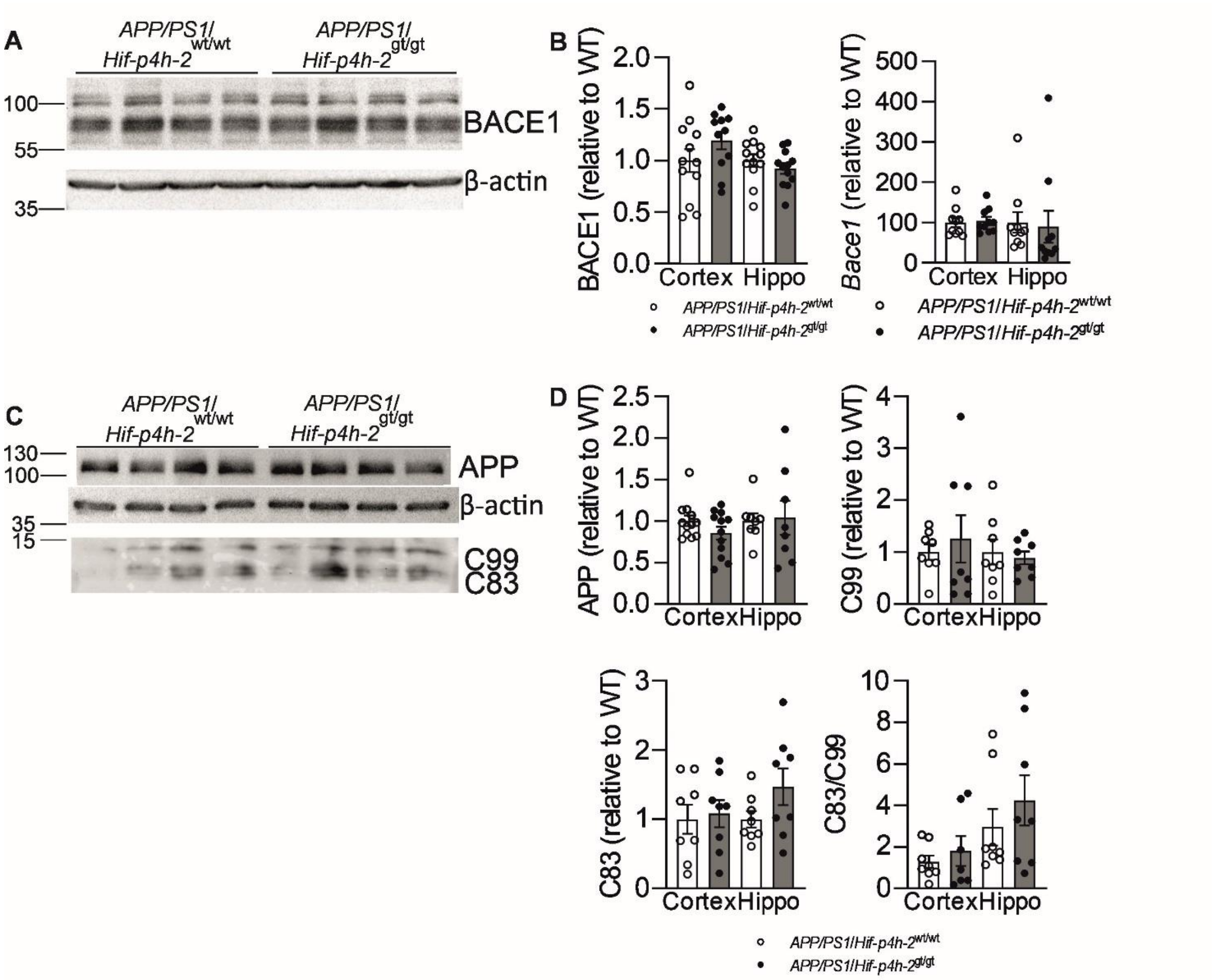

Supporting Figure 2. HIF-P4H-2 deficiency does not change BACE1 levels or APP processing in *APP/PS1* mice. (**A**) A representative Western blot of cortical BACE1 of 12-month-old HIF-P4H-2 wild-type (wt/wt) and deficient (gt/gt) *APP/PS1* mice and cortical and hippocampal mRNA levels of *Bace1*. β-actin was used as a loading control. (**B**) Quantification of BACE1 protein levels on a Western blot and *Bace1* mRNA leves in cortex and hippocampus. (**C**) A representative Western blot of APP and its C-terminal fragmentation (C99, C83). (**D**) Quantification of APP and its C-terminal fragments. WT, wild type; Hippo, hippocampus. Data are presented as mean ± standard error of mean.

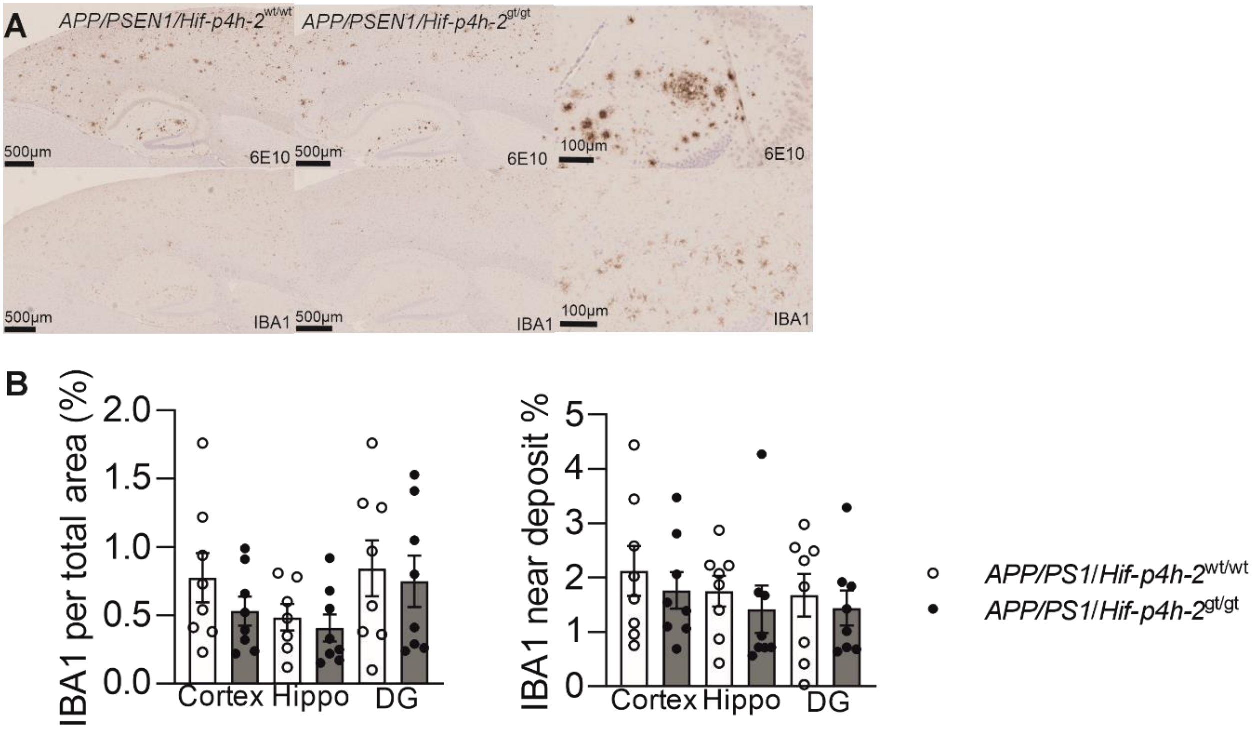

Supporting Figure 3. Microglial activation is not changed by HIF-P4H-2 deficiency in *APP/PS1* mice. (**A**) A representative immunohistochemical staining of β-amyloid (6E10) and microglia (IBA1) of 12-month-old HIF-P4H-2 wild-type (wt/wt) and deficient (gt/gt) *APP/PS1* brain. (**B**) Quantification of total IBA1 and IBA1 located near (50 µm) Aβ deposits in cortex, entire hippocampus (hippo) and *dentate gyrus* (DG). Data are presented as mean ± standard error of mean.

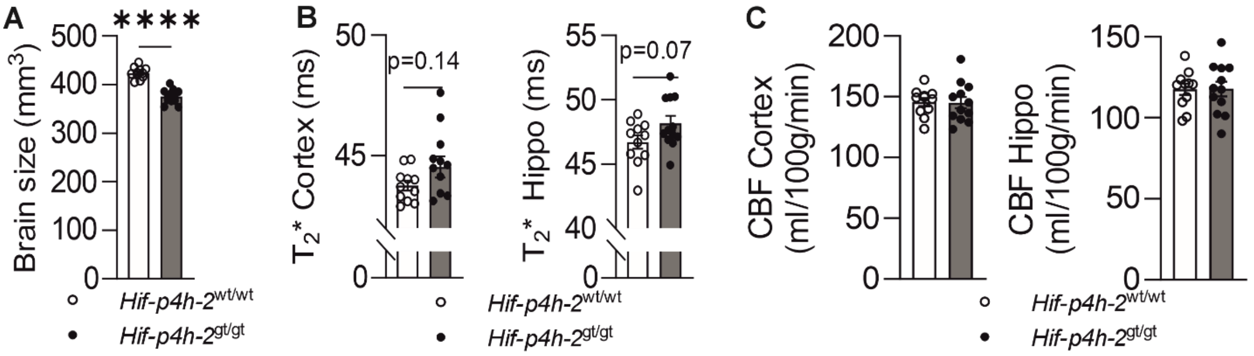

Supporting Figure 4. HIF-P4H-2 deficiency does not change cerebral blood flow in young mice. (**A**) The brain size of 2-month-old HIF-P4H-2 wild-type (wt/wt) and deficient (gt/gt) mice. (**B**) The T_2* time measured by magnetic resonance imaging (MRI) in cortex and hippocampus (hippo). (**C**) Cerebral blood flow (CBF) measured by MRI in cortex and hippocampus. Data are presented as mean ± standard error of mean. ****P < 0.0001 in two-tailed Student's t-test.

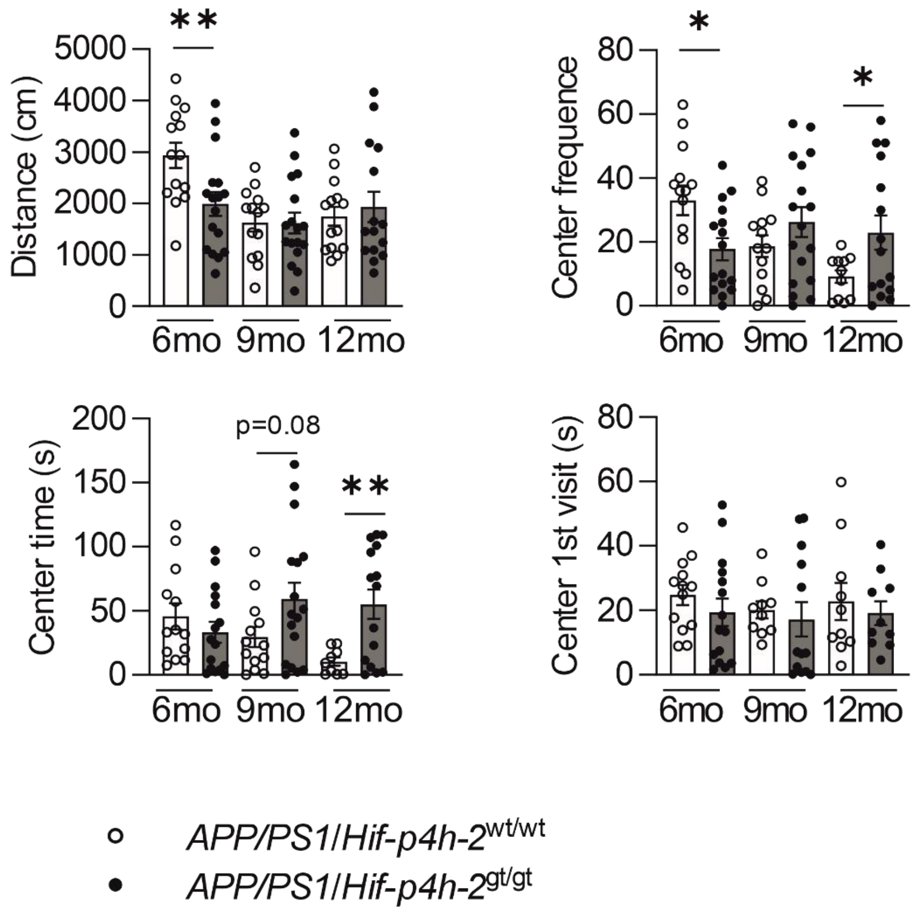

Supporting Figure 5. HIF-P4H-2-deficient *APP/PS1* mice maintain their behavioral activity upon aging in an open field test. Distance, center frequence, center time and time of the first visit to the center of HIF-P4H-2 wild-type (wt/wt) and deficient (gt/gt) *APP/PS1* mice measured in an open field test at 6, 9 and 12 months of age. Data are presented as mean ± standard error of mean. $^{*}P < 0.05$, $^{**}P < 0.01$ in two-way ANOVA.

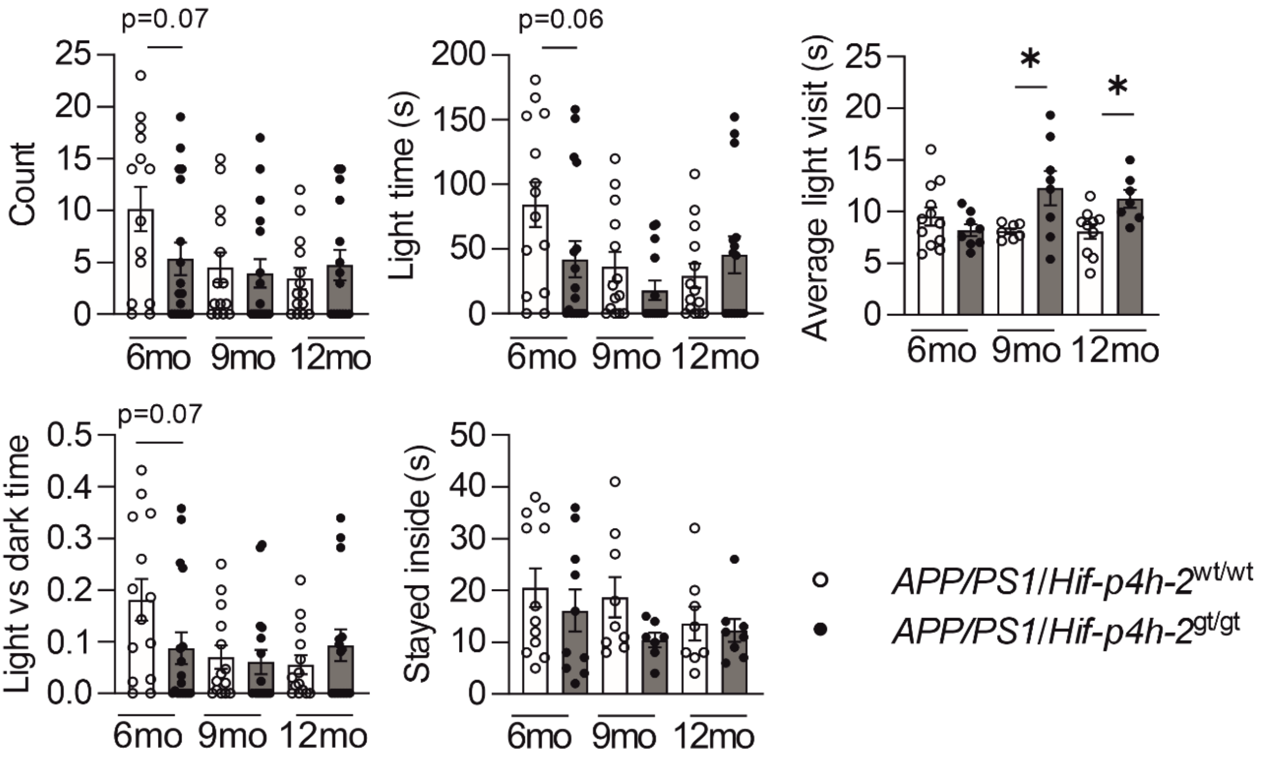

Supporting Figure 6. HIF-P4H-2 deficient *APP/PS1* mice maintain their behavioral activity upon aging in a dark/light test. Number of times visited (count), duration of total (light time) and individual (average light visit) visits to the light side, ratio of time spent in the light and dark side (light vs dark time), and time spent in the dark side before the 1st visit (stayed inside) of HIF-P4H-2 wild-type (wt/wt) and deficient (gt/gt) *APP/PS1* mice in the dark/light test at 6, 9 and 12 months of age. Data are presented as mean ± standard error of mean. **P* < 0.05 in two-way ANOVA.
